# Assessing a Novel 3D Assay System for Drug Screening against OS Metastasis

**DOI:** 10.3390/ph14100971

**Published:** 2021-09-25

**Authors:** Natalie Koons, Nicole Amato, Scott Sauer, David Warshawsky, Dalit Barkan, Chand Khanna

**Affiliations:** 1 Surgery Department, Massachusetts Veterinary Referral Hospital, Woburn, MA 01801, USA; namato@ethosvet.com; 2 Vuja De Sciences, Inc., Natick, MA 01760, USA; dw@vujade-life.com; 3 Department of Human Biology and Medical Sciences, University of Haifa, Haifa 3498838, Israel; dalitbrk@gmail.com; 4 Ethos Discovery, Woburn, MA 01801, USA; ckhanna@ethosvet.com

**Keywords:** osteosarcoma, metastatic endurance, small molecule inhibitors, oclacitinib, regorafenib, saracatinib, basement membrane extract assay

## Abstract

Osteosarcoma (OS) is an aggressive mesenchymal cell tumor that carries a poor long-term prognosis. Despite definitive surgery for the primary tumor and adjuvant chemotherapy, pulmonary metastasis is common and is the primary cause of morbidity. To improve outcomes for patients, we have developed and optimized a phenotypic screen for drugs that may target OS disseminated tumor cells (DTCs) and inhibit their metastatic outbreak rather than merely screening for cytotoxic activity against proliferating cells, as is commonly conducted in conventional drug discovery approaches. We report on the validation of a previously described 3D reconstituted basement membrane extract (3D BME) model system for tumor dormancy and metastatic outgrowth adapted to clonal pairs of high and low metastatic OS cells. A post-hoc validation of the assay was possible by comparing the activity of a drug in our assay with early evidence of activity in human OS clinical trials (regorafenib and saracatinib). In this validation, we found concordance between our assay and human clinical trial experience We then explored an approved veterinary small molecule inhibitor of Janus kinase-1 (oclacitinib) as a potential drug candidate to take advantage of the high prevalence of OS in pet dogs and its translational value to humans. Despite the biological rationale, we found no evidence to support the use of oclacitinib as an antimetastatic agent in OS. The findings support our 3D BME assay as a highly efficient method to examine drugs for activity in targeting OS DTCs.

## 1. Introduction

Osteosarcoma (OS) is a neoplasia of mesenchymal cell origin. The aggressive nature of OS results in poor prognoses, with metastasis to the lungs being the most common cause of death. Despite complete resection of the primary tumor and adjuvant chemotherapy for patients with localized disease, patients (~35%) develop recurrent metastases most commonly to the lungs within a few years and the five-year survival rate is less than 30% [1,2]. Accumulating evidence in the literature suggests that occurrence of cancer years after initial treatment arises from disseminated tumor cells (DTCs) that have colonized distant organs such as the lungs and remained dormant (quiescent) [3]. These dormant DTCs do not respond to chemotherapy or radiotherapy treatments which target actively dividing cells, and they cannot be detected with current diagnostic imaging, thus lingering in the body as ticking time bombs [3]. Therefore, the continuous presence of these dormant DTCs even after primary treatment may explain metastatic recurrence years after initial diagnosis. To date, no substantive improvements in outcome have been seen in these patient cohorts in over 30 years. Hence, a change in drug discovery paradigms to develop drugs that address the unmet needs of patients is critical [4].

The current approaches to cancer drug development prioritize drugs that kill rapidly dividing cells or induce apoptosis in cancer cells. Although these targets are characteristics of cancer, they do not adequately represent the biology of DTC metastatic progression. DTCs undergo adaptation, survival, and enter a phase of dormancy and eventually outgrowth at their metastatic site. This multistep process which we coined metastatic endurance (ME) [4] is just beginning to be addressed by cancer drug development. Hence, new assays defining the activity of drugs that target DTC ME may be adapted for use in OS.

Here, we describe the adaptation of a previously reported 3D reconstituted basement membrane extract (3D BME) system modeling breast cancer tumor dormancy and outgrowth [5,6] to model ME of low and high metastatic OS cells. Clonally related OS cell line pairs with validated high and low metastatic potential in mice and other assays of metastasis were used [5]. We then examined drugs that have been used in human OS trials for their ability to block metastatic progression. Small molecule inhibitors investigated include saracatinib (a Src kinase inhibitor that appears to have failed in human OS trials [7]; negative control), regorafenib (a multi-targeting kinase inhibitor that appears to have therapeutic activity in early human OS trials [8]; positive control), and an investigational drug oclacitinib (a Janus kinase (JAK) inhibitor approved for use in canine atopic dermatitis [9]) to test its activity against OS metastasis.

We first identified exposures of each drug that had minimal cytotoxic effect on two highly metastatic OS cell lines in conventional 2D conditions (i.e., grown as a monolayer on plastic). We then used exposures of these drugs well below a cytotoxic threshold in the 3D BME, to identify drugs that may have selective activity against OS ME. Our primary objective was to determine if the 3D BME assay would recapitulate early human clinical trial results. Our secondary objective was to determine if oclacitinib, a currently FDA-approved veterinary drug (Apoquel^®^), would exert a desired antimetastatic effect in 3D BME. Our hypothesis was that oclacitinib would suppress ME within OS cell lines.

Our results indicate that the adapted 3D BME assay could create a model phenocopy of previously characterized clonally related OS cells that differ in metastatic proclivity in mouse models. Interestingly, the selected agent with promising human clinical trial activity (regorafenib) was active in the adapted 3D BME system, and the selected agent without measurable activity in human OS trials (saracatinib) was inactive in the adapted 3D BME system. The FDA-approved veterinary drug (oclacitinib) was not found to be active despite a hypothesized biological rationale in cancer metastasis. While our data suggests against prioritizing this drug for further development in OS, we believe the 3D BME system and other assays that model metastatic progression will uncover new therapeutics with activity against ME that are amenable to the proposed cross-species approach to metastasis drug development in OS.

## 2. Results

### 2.1. Validation of Matched High and Low Metastatic OS Cell Pairs in the 3D BME Assay

**Figure 1:**
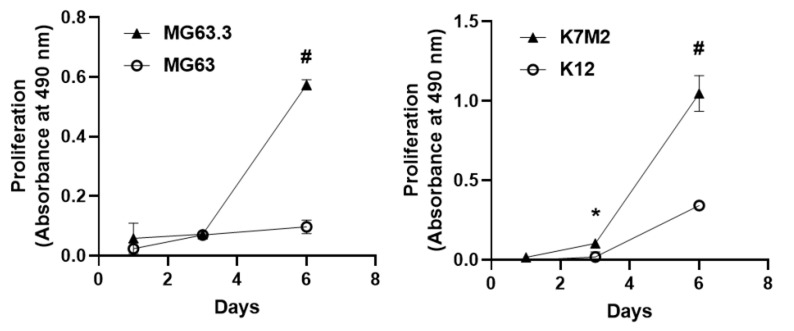
Comparison of growth kinetics of high and low metastatic potential OS cells in the 3D BME system. Growth kinetics of matched human (MG63—low; MG63.3—high) and murine (K12—low; K7M2—high) osteosarcoma cells were observed over a 6-day period in the 3D BME system. Matched cells were plated under the same conditions and assessed for proliferation on days 1, 3, and 6 using an MTS assay. Highly metastatic cells undergo outbreak after 3 days exhibited by the high rate of proliferation from days 3–6, while low metastatic lines were growth arrested or displayed low rates of proliferation. These graphs are from a single representative assay (replicates = 4) with n = 3 biological repeats with similar results (* *p* < 0.05; # *p* < 0.005).

Previously validated high and low metastatic human OS cell lines MG63 (low metastatic) and MG63.3 (high metastatic) cells [10] were cultured in the 3D BME system as described in Methods. Our results demonstrate that MG63 cells were growth arrested (dormant) (Figure 1 left panel) over the six-day experiment period (consistent with a nonmetastatic phenotype). Interestingly, MG63.3 displayed a transient growth arrest followed by exponential proliferation beginning at day 3 (consistent with a metastatic phenotype). Furthermore, the proliferation of the MG63.3 cell line at day 6 was significantly higher (# *p* < 0.005) compared to the MG63 cell line (Figure 1 left panel).

Similar results were found using a distinct and previously validated pair of murine OS cells, K12 (low metastatic) and K7M2 (high metastatic) cell lines [11]. K12 cells were growth arrested and resumed proliferation at day 3 (Figure 1 right panel). Similarly, K7M2 cells demonstrated an initial lag phase of proliferation followed by outbreak and exponential proliferation beginning on day three. However, the proliferation of K7M2 cells from day 3 forward was significantly higher compared to K12 cells (Figure 1 right panel) (* *p* ≤ 0.05; # *p* < 0.005).

### 2.2. Assessment of Direct Drug Cytotoxicity by Cell Number

**Figure 2:**
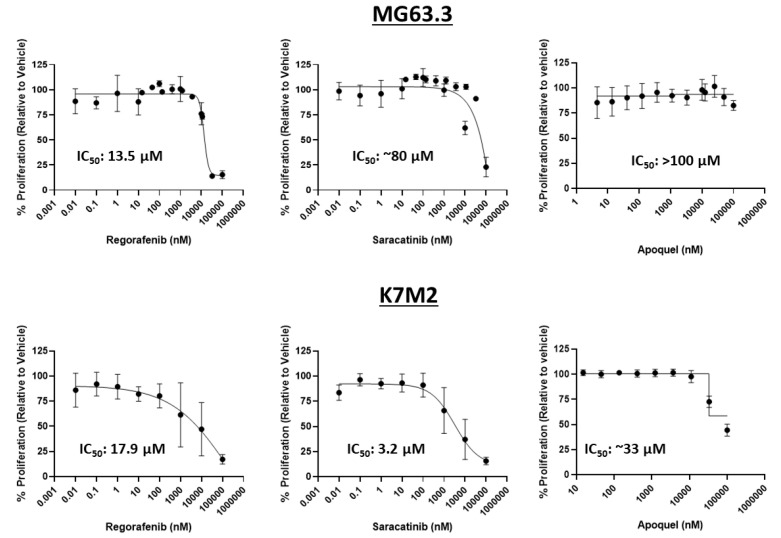
Drug exposures at or below 1 μM were determined to be non-cytotoxic in highly metastatic OS cells. The inhibition of OS cell number in 2D culture was measured in dose-response via MTT assay. Regorafenib, saracatinib, and oclacitinib were tested at various doses (10 pM-100 μM) in MG63.3 (top) and K7M2 (bottom) for 72 h. IC50s were calculated using a four-parameter variable slope nonlinear fit in GraphPad Prism. (N = 3, replicates = 3–9).

MTT assays were used to assess the influence of oclacitinib, saracatinib, and regorafenib on cell number in 2D monolayer conditions to determine the maximum physiological concentration that will not have cytotoxic activity on highly metastatic OS cells. This concentration will be further tested for inhibition of ME of highly metastatic OS cell lines in the 3D BME system. Collectively, exposures up to 1 μM were associated with minimal, consistent, and dose-dependent reductions in cell number across drugs and cell lines (Figure 2). Oclacitinib showed minimal inhibition of OS cell number in either cell line within physiologically relevant exposures up to 1 μM. Saracatinib and regorafenib showed no effect on remaining cell number over the assessed exposures in MG63.3 cells. Based on these MTT data, exposures of ≤1 μM were used in subsequent 3D BME experiments. This 1 μM exposure was also selected since such exposures are typically physiologically achievable for most small molecule inhibitors in human or preclinical studies. Accordingly, the antimetastatic activity of the drugs shown in the 3D BME assay (below 1 μM) may be described as independent of cytotoxicity.

### 2.3. Effect of Drug Treatment on OS Cells in 3D BME

**Figure 3:**
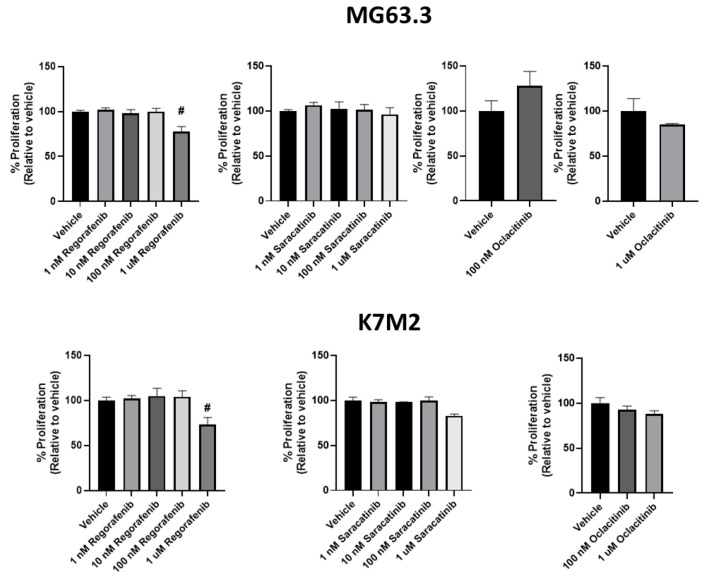
The activity of antimetastatic drugs in the 3D BME system on day 6. Using the 3D BME system, highly metastatic osteosarcoma cells were treated with regorafenib, saracatinib, and oclacitinib at varying doses (1 nM−1 μM) and the % proliferation at day 6 was determined by MTS assay. MG63.3 and K7M2 cells were treated with the inhibitors and only regorafenib was found to significantly inhibit the proliferation (relative to vehicle [0.1% DMSO] control) (>20%, # *p* < 0.005) across both cell lines at a dose (1 μM) that is not cytotoxic in 2D culture (See Figure 2).

The effect of regorafenib, saracatinib, and oclacitinib on ME of highly metastatic OS cell lines was next determined. Regorafenib significantly inhibited the proliferative outgrowth of both MG63.3 and K7M2 cell lines at physiologically relevant and non-cytotoxic exposures of 1 μM (Figure 3; ~20% inhibition in both cell lines, # *p* ≤ 0.005) compared to vehicle treated cells. Significant inhibition was not seen at 100 nM. Conversely, saracatinib did not significantly inhibit the proliferative outgrowth of both cell lines in the 3D BME system at or below 1 μM. Oclacitinib also had no significant effect in the 3D BME assay on the proliferative outgrowth in either cell line tested (Figure 3).

## 3. Discussion

This study was performed to validate a 3D BME assay using paired low and high metastatic OS cell lines and then assess efficacy of drugs against metastatic endurance (ME). Two small molecular kinase inhibitors, regorafenib and saracatinib were chosen as suspected positive and negative controls respectively given their previous clinical results in early human clinical trials. The results with these two “control drugs” provide a post hoc validation of the 3D BME assay as a predictive screen for drugs with antimetastatic activity in this adaptation of the 3D BME assay. A third small kinase inhibitor was then assessed for efficacy against ME in OS cells.

The experiments performed here included OS cell lines that have been rigorously evaluated for biologic and metastatic behavior [10,11]. The selection of these cell lines in a phenotypic drug screen has some inherent weakness over well-characterized patient-derived models (PDXs) since the three drugs tested are targeted agents and the expression of targets is not known, not conserved, and not likely relevant for all cell models. Nonetheless, the value of a phenotypic screen is the opportunity to identify drugs or therapeutic candidates with the desired phenotype of blocking metastatic potential independent of target knowledge. It is reasonable to ask if two highly metastatic cell lines are sufficient for such a screen and if the addition of a canine OS cell line would add value to a screen that aspires to evaluate promising drugs prior to clinical trials for pet dogs with OS. The addition of more cell lines to our screen is part of ongoing studies; nonetheless, we do not believe there is significant value to the addition of a canine cell line, per se. Canine and human OS cells share many dysregulated intracellular signaling pathways, allowing a human OS cell line (MG63.3) to reasonably represent canine disease [12]. The recognized genomic complexity and heterogeneity of OS, and our dependence on highly characterized cell lines is likely a greater weakness to the current approach than the absence of a canine cell line. Additional cells with known metastatic potential from human patient derived xenograft (PDX) models and genetically engineered models (GEMs) will be part of a planned assay expansion. Our approach to using MTT to identify exposures for subsequent 3D BME testing was intended to identify drug exposures that were not directly cytotoxic and that would not confound our assessment of inhibition of ME. In parallel, we aligned the non-cytotoxic exposures with exposures of these drugs that are physiologically attainable in human clinical trials or preclinical models. This approach is reasonable and necessary; however, we are aware of the challenges associated with pharmacokinetic modeling from cell-based assays to the in vivo setting. While our proposal uses the OS modified BME assay to identify drugs that may target important steps in the metastatic cascade, it cannot fully recapitulate all facets of metastasis. To confirm our results, all findings using the OS modified BME assay will be confirmed in complimentary ex vivo and/or in vivo metastasis studies.

One additional challenge associated with our 3D BME assay approach is the exclusion of the innate/adaptive immune system’s influence on the metastatic niche [3]. Only the impact of the extracellular matrix and nutritional stressors placed upon the metastatic cancer cells are modeled here [3], and, as such, the assay does not mimic the interaction of the dormant tumor cell with an active immune system. It is important to recognize and address this limitation. Inactivity against OS cells in 3D BME should not be the sole criterion that eliminate potential therapeutic candidates but may be a valuable approach to force rank or prioritize candidates in the setting of resource or clinical trial patient scarcity. We plan to expand our screening assays to include models such as PuMA (pulmonary metastasis assay) which models the lung metastatic niche [13]. In light of our planned assay expansion, 3D BME becomes an important and higher-throughput screening step to eliminate inactive candidates from further testing in the more labor and resource intensive, but physiologically relevant, ex vivo PuMA model. The added efficiency provided by this intermediate step is highlighted by the large number of drugs that can be screened in parallel.

Regorafenib, our positive control, significantly suppressed outgrowth of cells in 3D BME at clinically relevant doses, suggestive of potential antimetastatic activity. Regorafenib is already used across a multitude of cancer indications including hepatocellular carcinoma, renal cell carcinoma, and gastrointestinal stromal cell tumors [14,15,16]. It recently showed early promise in a phase two human clinical trial for OS and soft tissue sarcoma, where progression of metastasis had occurred in the face of traditional cytotoxic chemotherapies (REGOSARC, REGOBONE) [17,18], hence, its use in our study as a positive clinical control. Regorafenib suppresses multiple receptors involved in angiogenesis (VEGFR1,2,3, TIE2, and PDGFR-B). It also has demonstrated inhibitory effects on oncogenic kinases (KIT and RET) [19]. One shortcoming of its use as a positive control is that this drug has not been proven to work alone at blunting ME in the clinical setting. In the previously mentioned REGOBONE clinical trial, the patient population had advanced metastatic disease and had all been previously treated with conventional cytotoxic agents. This combination of therapies is not recreated in our assay system. Additionally, patients with naturally occurring disease may have different subtypes of OS than the cell lines used in our study. Nonetheless, the results are in accordance with previous clinical trials and thus supports our approach.

Saracatinib was used as a negative control for this study given its poor performance in a clinical trial in humans with progressive OS. While the clinical trial showed an increase in progression free survival there was no apparent benefit to long term overall survival [7]. It is fair to conclude that the question about saracatinib activity against OS metastasis was never answered in the referenced clinical trial as a result of insufficient patient accrual. Enrollment and median overall survival times were not reached for the experimental or placebo group. Nonetheless, the totality of data from the small number of evaluable patients is sufficient to conclude that saracatinib was not associated with a profound treatment benefit. Interestingly, our data also suggests saracatinib is an inactive antimetastatic drug in OS.

Oclacitinib, our drug of interest, is a Janus kinase inhibitor approved for use in controlling allergy, inflammation, and pruritus in dogs. The drug binds to JAK1/2/3 and TYK2 [9] with its most potent inhibitory action against JAK1 (1.8 times that of JAK2 and 9.9 times that of JAK3). Given that increased JAK2/STAT3 activity has been documented in MG63 OS cells with lung metastasis [20] and that upregulation of STAT3 in OS cells have shown to promote invasion and metastasis [21], it was reasonable to ask whether oclacitinib would inhibit escape from dormancy in OS cell lines. Based on our findings, oclacitinib was not useful at suppressing metastatic progression in OS in vitro. The biologic dependency of the JAK/STAT pathway in MG63.3 or K7M2 is not known. The pathway may not be one of the crucial steps in escape from dormancy in OS cells or it may be part of a highly redundant biology that was compensated for by other pathways upon its blockade. Nonetheless, oclacitinib should not be prioritized above other agents for clinical development. An additional potential advantage in choosing oclacitinib for our study was the opportunity to accelerate translation/repurposing of this FDA-approved veterinary drug to treat human OS metastasis should it have proven active. Unfortunately, our results indicate that oclacitinib was not active in suppressing ME in two distinct highly metastatic OS cell lines. Given the consideration that there was no biologically useful activity with this drug as compared to regorafenib, we will not prioritize further development of oclacitinib in OS metastasis at this time.

An additional consideration in deprioritizing oclacitinib as a therapeutic candidate is concern for its immunosuppressive properties. In initial safety studies, concern was raised about emergence of diseases commonly seen in immunosuppressed dogs (e.g., demodicosis) [22]. Subsequent in vitro studies have shown oclacitinib has pro-apoptotic effects on T cells [23]. In light of the known importance that T cell activity exerts on regulating tumor outgrowth within the metastatic microenvironment, [3,24] one may argue that inhibition of T cell activity while trying to control metastatic progression is counterproductive.

Despite oclacitinib’s lack of efficacy reported here, we believe the described 3D BME assay approach has value as part of a phenotypic screen of cancer metastasis, that in this case could be rapidly applied to in vivo validation studies in canine OS as a model for human OS. The switch from a dormant cell to an active metastatic cell involves multiple intracellular pathways [8,25] and so it is perhaps not surprising oclacitinib, a drug that selectively targets the JAK/STAT pathway, proved ineffective. The success noted with regorafenib, which targets multiple pathways, highlights the potential need for probing combination therapies. Using the 3D BME assay system, screening for additive or even synergistic combinations of multiple small molecule inhibitors (or small molecule inhibitors in combination with classic cytotoxic chemotherapies) is feasible and efficient. Once identified, successful combinations of already approved FDA drugs can be rapidly translated to clinical trials in dogs or humans directly.

## 4. Materials and Methods

### 4.1. Cell Culture

Two previously described clonally related and biologically validated high and low metastatic OS cell lines MG63.3 (highly metastatic; human OS), MG63 (low metastatic, human OS), K7M2 (highly metastatic murine OS), and K12 (low metastatic murine OS), [10] were used to optimize and validate the 3D BME assay for use with OS cells. The highly metastatic clone from each pair was then used to assess antimetastatic activity of the selected drugs.

Cells were maintained and passaged in Dulbecco’s modified Eagle medium (DMEM) +4.5 g/L D-Glucose and 2 mM L-glutamine supplemented with 10% fetal bovine serum (FBS) and 1% penicillin/streptomycin (P/S) (Gibco, Grand Island, NY, USA), and incubated at 37 °C in 5% humidified CO_2_.

Oclacitinib, saracatinib, and regorafenib (Selleck Chemical, Houston, TX, USA) were each dissolved in DMSO at a stock concentration of 10 mM.

### 4.2. Assessment of Cell Number and Cytotoxic Activity of Each Drug in 2D Culture

The number of viable cells present in 2D culture was determined by MTT as previously reported [26]. Briefly, cells were plated in 96-well plates at 2000 cells/well and incubated at 37 °C for 24 h. After 24 h, the media was replaced with fresh media containing drugs at the desired concentrations (10 pM−10 μM) and incubated at 37 °C for 72 h. After 72 h, MTT reagent (20 μL/well of a 5 mg/mL stock solution in sterile water; Sigma, St. Louis, MO) was added to each well and the plate was incubated at 37 °C for 2 h. DMSO (100 μL/well) was then added to each well to solubilize the formazan crystals.

The resulting absorbance was measured at 490 nm via plate reader (Molecular Devices, San Jose, CA, USA). DMSO (0.1%) was used as vehicle control, while 20 μM rapamycin was used as a positive cell-kill control [27,28]. The experimental conditions were conducted in triplicate and each experiment was repeated three times. Regression lines and IC50s were calculated using a four-parameter variable slope nonlinear fit in GraphPad Prism.

### 4.3. Assesment of ME and Drug Response of OS Cells in the 3D BME System

The influence of drug exposure on the transition from tumor dormancy to metastatic growth of OS cells was assessed in a 3D BME system as described before [5,29].

Cultrex^®^ PathClear, Reduced Growth Factor Basement Membrane Extract (BME—Bio-Techne, Minneapolis, MN, USA) was added (50 μL/well) to a 96-well plate and allowed to gel by incubating at 37 °C for ≥30 min. Cells were harvested, washed with DMEM (no glucose, no FBS, Gibco, Grand Island, NY, USA), and centrifuged at 1500× *g* 5 min. The media was aspirated, and the remaining cell pellet flicked for ~10 s to ensure disaggregation.

Cells were diluted in assay media containing 2% BME, resuspended vigorously, and 150 μL of the cell suspension was added on top of the gelled 3D BME layer. For comparison, matched low-metastatic cell lines (MG63 and K12) were grown under the same conditions as the highly metastatic cells (MG63.3 and K7M2) and did not exhibit the same growth rate or kinetic profile, highlighting the differences in their metastatic potential. Proliferation was determined in the control vehicle (cells in media + 0.1% DMSO) or treated wells at indicated days by the addition of 20 μL/well of MTS reagent (Cell Titer 96 Aqueous One, Promega, Madison, WI, USA), followed by incubation at 37 °C for 2 h and absorbance measurements at 490 nm via plate reader (Molecular Devices, San Jose CA, USA).

### 4.4. Statistical Analysis

All conditions were conducted in quadruplicate and each experiment was carried out at least 3 separate times. All comparisons were made between treatment and vehicle control at concordant time points. Statistical analyses and non-linear regressions were derived using GraphPad Prism statistical software.

## 5. Conclusions

The results reported here validate a modified 3D BME assay system for use with OS cells to assess efficacy of drugs or therapeutic agents against ME satisfying our primary objective. We showed that the assay system was effective at mirroring previously known clinical results with two drugs: regorafenib and saracatinib. In 3D BME, regorafenib suppressed metastatic outgrowth of two highly metastatic OS cell lines while saracatinib was ineffective. Oclacitinib also proved ineffective at suppressing ME in two highly metastatic cell lines, refuting our working hypothesis. Next steps include expansion of the number of cell lines used in 3D BME testing, and continued examination of drugs with potential for translation to anti-metastatic agents. Once identified, these agents will be further evaluated for efficacy against ME using the ex vivo pulmonary metastasis assay to better model an in vivo metastatic niche. Drugs found to show continued efficacy against ME in the PuMA may be brought forward for clinical trials, specifically trials in the canine OS population as a model for human OS.

## Data Availability

Data is contained within the article.
